# Shifts in taxonomic and functional microbial diversity with agriculture: How fragile is the Brazilian Cerrado?

**DOI:** 10.1186/s12866-016-0657-z

**Published:** 2016-03-16

**Authors:** Renata Carolini Souza, Iêda Carvalho Mendes, Fábio Bueno Reis-Junior, Fabíola Marques Carvalho, Marco Antonio Nogueira, Ana Tereza Ribeiro Vasconcelos, Vânia Aparecida Vicente, Mariangela Hungria

**Affiliations:** Embrapa Soja, Soil Biotechnology, C.P. 231, 86001-970 Londrina, PR Brazil; Department Microbiology, Universidade Federal do Paraná, C.P. 19031, 81531-990 Curitiba, PR Brazil; Embrapa Cerrado, Soil Microbiology, C.P. 08223, 73301-970 Planaltina, DF Brazil; LNCC, Labinfo, Av. Getúlio Vargas 333, 25651-071 Petrópolis, RJ Brazil

**Keywords:** Shotgun metagenome, Soil microbiome, Functional biodiversity, Soil management, No-tillage, Cerrado

## Abstract

**Background:**

The Cerrado—an edaphic type of savannah— comprises the second largest biome of the Brazilian territory and is the main area for grain production in the country, but information about the impact of land conversion to agriculture on microbial diversity is still scarce. We used a shotgun metagenomic approach to compare undisturbed (native) soil and soils cropped for 23 years with soybean/maize under conservation tillage—“no-till” (NT)—and conventional tillage (CT) systems in the Cerrado biome.

**Results:**

Soil management and fertilizer inputs with the introduction of agriculture improved chemical properties, but decreased soil macroporosity and microbial biomass of carbon and nitrogen. Principal coordinates analyses confirmed different taxonomic and functional profiles for each treatment. There was predominance of the Bacteria domain, especially the phylum Proteobacteria, with higher numbers of sequences in the NT and CT treatments; Archaea and Viruses also had lower numbers of sequences in the undisturbed soil. Within the Alphaproteobacteria, there was dominance of Rhizobiales and of the genus *Bradyrhizobium* in the NT and CT systems, attributed to massive inoculation of soybean, and also of Burkholderiales. In contrast, *Rhizobium*, *Azospirillum, Xanthomonas, Pseudomonas* and *Acidobacterium* predominated in the native Cerrado. More Eukaryota, especially of the phylum Ascomycota were detected in the NT. The functional analysis revealed lower numbers of sequences in the five dominant categories for the CT system, whereas the undisturbed Cerrado presented higher abundance.

**Conclusion:**

High impact of agriculture in taxonomic and functional microbial diversity in the biome Cerrado was confirmed. Functional diversity was not necessarily associated with taxonomic diversity, as the less conservationist treatment (CT) presented increased taxonomic sequences and reduced functional profiles, indicating a strategy to try to maintain soil functioning by favoring taxa that are probably not the most efficient for some functions. Our results highlight that underneath the rustic appearance of the Cerrado vegetation there is a fragile soil microbial community.

**Electronic supplementary material:**

The online version of this article (doi:10.1186/s12866-016-0657-z) contains supplementary material, which is available to authorized users.

## Background

The Cerrado region represents the second largest biome of the Brazilian territory, with an area of approximately 2 million km^2^ [1]. The soils are rich in aluminum, poor in nutrients and very acidic, supporting only an adapted vegetation typically composed of a gradient of grassland, savannah and forest, interspersed with riparian or gallery forests, patches of semi-deciduous forest, swamp and marshes [2]. However, with appropriate chemical correction, Cerrado soils can be very productive, and since the early 1960s large areas have been incorporated into agriculture [3], such that currently the region represents the main grain producing area in Brazil.

Soils are the more diverse environment in terms of microorganisms on Earth, with approximately 1,000 Gbp of microbial genome sequences per g of soil [4]. Microorganisms directly affect the environment and agricultural systems, by means of an array of mechanisms that include biological nitrogen fixation [5], suppression of diseases [6], decomposition of organic matter [7], plant growth promotion [8], soil nutrient cycling [9] and bioremediation [10]. However, soil microbial community structure and its associated biological processes can be readily affected by land use, as a result of changes in soil structure, water holding capacity, temperature fluctuations, organic matter and nutrients contents, pH, introduction of new plant species, and agrichemical inputs (e.g. [3, 6, 8]).

For decades, several studies have measured the impact of agriculture on soil microorganisms diversity and function, but using limited methodologies that identified few microorganisms and/or detected only generalist activities or microbial biomass (e.g. [11–13]). Soil metagenome studies are finally revealing how deep the impacts of anthropogenic action may be. For example [14], confirmed that native forest soils had higher bacterial diversity than agricultural soils, while [15] showed greater relative abundance of certain bacterial orders and Archaea in a soil under conservation management, in comparison to another on which conventional practices had been adopted.

Brazilian economy greatly relies on agriculture, but the media frequently claims that the country adopts non-sustainable practices in agriculture. Considering soil microbial biomass, the Brazilian Cerrado is even more sensitive than the Amazon to the introduction of agriculture [13], raising concerns about the impact on microbial community. Limitations of using specific methodologies or genes rely on bias of specific primers, detection of uncultivable microorganisms, among others, but great advances have been achieved with the metagenomic shotgun approach, opening opportunities for revealing genetic and metabolic diversity as well as new metabolic routes, genes and products (e.g. [15–19]). Therefore, in order to better understand the impact of agriculture on the Cerrado soils, we used a shotgun metagenomic approach with taxonomic and functional analyses, comparing undisturbed and cropped areas.

## Results

### Soil physical and chemical properties and classical microbiological parameters

When physical properties were addressed, we observed that 23 years of continuous cropping resulted in increased microporosity and decreased macroporosity, for both the NT and CT treatments. In addition, soil density was increased in the CT system (Table 1).

**Table 1:** Soil physical proprieties at the 0–10 cm layer in an Oxisol under native vegetation of Cerrado (Native) or cropped with soybean/corn under no-tillage (NT) or conventional tillage (CT) systems

*Physical*				
*Treatment*	*Total porosity*	*Microporosity*	*Macroporosity*	*Density*
	*m* ^*3*^ */m* ^*3*^	*m* ^*3*^ */m* ^*3*^	*m* ^*3*^ */m* ^*3*^	*Mg/m* ^*3*^
NT	0.59 A	0.41 A	0.17 B	0.91 B
CT	0.59 A	0.44 A	0.15 B	0.95 A
Native	0.60 A	0.39 B	0.21 A	0.91 B
*p*	0.2022	0.0000	0.0000	0.0000

Values in columns sharing the same letter do not differ significantly (*p* < 0.05) as determined by the Tukey’s test

For the chemical properties, the results obtained in the undisturbed treatment highlight the typical properties of the Cerrado, with high Al content and acidity, low P and nutrients (Tables 1 and 2). Soil liming and fertilizer inputs to the cropped area increased the level of nutrients, especially P, and increased pH. In comparison to the native undisturbed area, organic matter slightly decreased with cropping under the conservationist system of NT, with a further significant decrease in the CT (Table 2).

**Table 2:** Soil chemical proprieties at the 0–10 cm layer in an Oxisol under native vegetation of Cerrado (Native) or cropped with soybean/corn under no-tillage (NT) or conventional tillage (CT) systems

*Chemical*								
*Treatment*	*Al – exchangeable (titrimetry)*	*Ca (atomic absorption)*	*H + Al - Acidity (titrimetry)*	*K (flame photometer)*	*Organic matter (OM) (Walkley & Black)*	*Mg (atomic absorption)*	*pH in water*	*P (Mehlich1 - spectrophotometry)*
	*me/100 cc*	*me/100 cc*	*me/100 cc*	*mg/L*	*%*	*me/100 cc*	*pH*	*mg/L*
NT	0.007 B	2.847 A	5.745 B	46.667 A	3.209 AB	0.978 A	5.670 A	26.563 A
CT	0.014 B	1.545 B	4.717 C	30.000 B	2.751 B	0.556 B	5.647 A	10.417 B
Native	0.682 A	0.093 C	8.432 A	44.667 A	3.667 A	0.142 C	4.687 B	0.180 C
*p*	0.0000	0.000	0.0003	0.0118	0.0262	0.0001	0.0001	0.0008
CV%	12.68	9.39	4.94	9.61	7.68	9.31	1.20	22.37

Values in columns sharing the same letter do not differ significantly (*p* < 0.05) as determined by the Tukey’s test

A three-fold decrease in soil MB-C and MB–N was observed with agriculture introduction in the Cerrado (Table 3). The arylsulfatase and acid phosphatase activities of the native area resembled those of the NT, but a decrease was verified in the CT treatment. β-glucosidase was also highest in the NT treatment, but it’s activity in the CT was similar to that observed in the native Cerrado (Table 3).

**Table 3:** Microbial enzymes activities and microbial biomass of carbon and nitrogen evaluated in the soils samples at the 0–10 cm layer in an Oxisol under native vegetation of Cerrados (Native) or cropped with soybean/corn under no-tillage (NT) or conventional tillage (CT) systems

*Treatment*	*MB-N	*MB-C	*β-Glucosidase*	*Arylsulfatase*	*Acid Phosphatase*
	*mg/kg soil*	*mg/kg soil*	*mg p-nitrofenol/kg soil/h*		
NT	30.71 B	215.86 B	150.03 A	70.73 A	812.09 A
CT	23.85 B	150.65 C	92.51 B	41.75 B	621.13 B
Native	84.13 A	539.08 A	94.30 B	71.50 A	824.58 A
*p*	0.0017	0.0000	0.0124	0.0126	0.0061
CV%	18.19	3.34	12.63	12.04	5.41

Values in columns sharing the same letter do not differ significantly (*p* < 0.05) as determined by the Tukey’s test

*MB-N- Microbial Biomass-Nitrogen

*MB-C- Microbial Biomass-Carbon

### Sequencing analyses

In the shotgun metagenomic approach, for each treatment about 5 million sequences were generated, resulting in 49.2 million reads and 1.31 x 10^10^ bp. When submitted to the MG-RAST server, an average of 2.23 million proteins was classified as known proteins and 2.98 million as predicted proteins but with unknown function (Additional file 1: Table S1).

### Rarefaction curves and principal coordinates analysis

The rarefaction curves from samples generated in MG-RAST showed that even with almost 50 million sequences, the curves were not saturated, indicating high genetic diversity (Additional file 2: Figure S1).

**Fig. 1:**
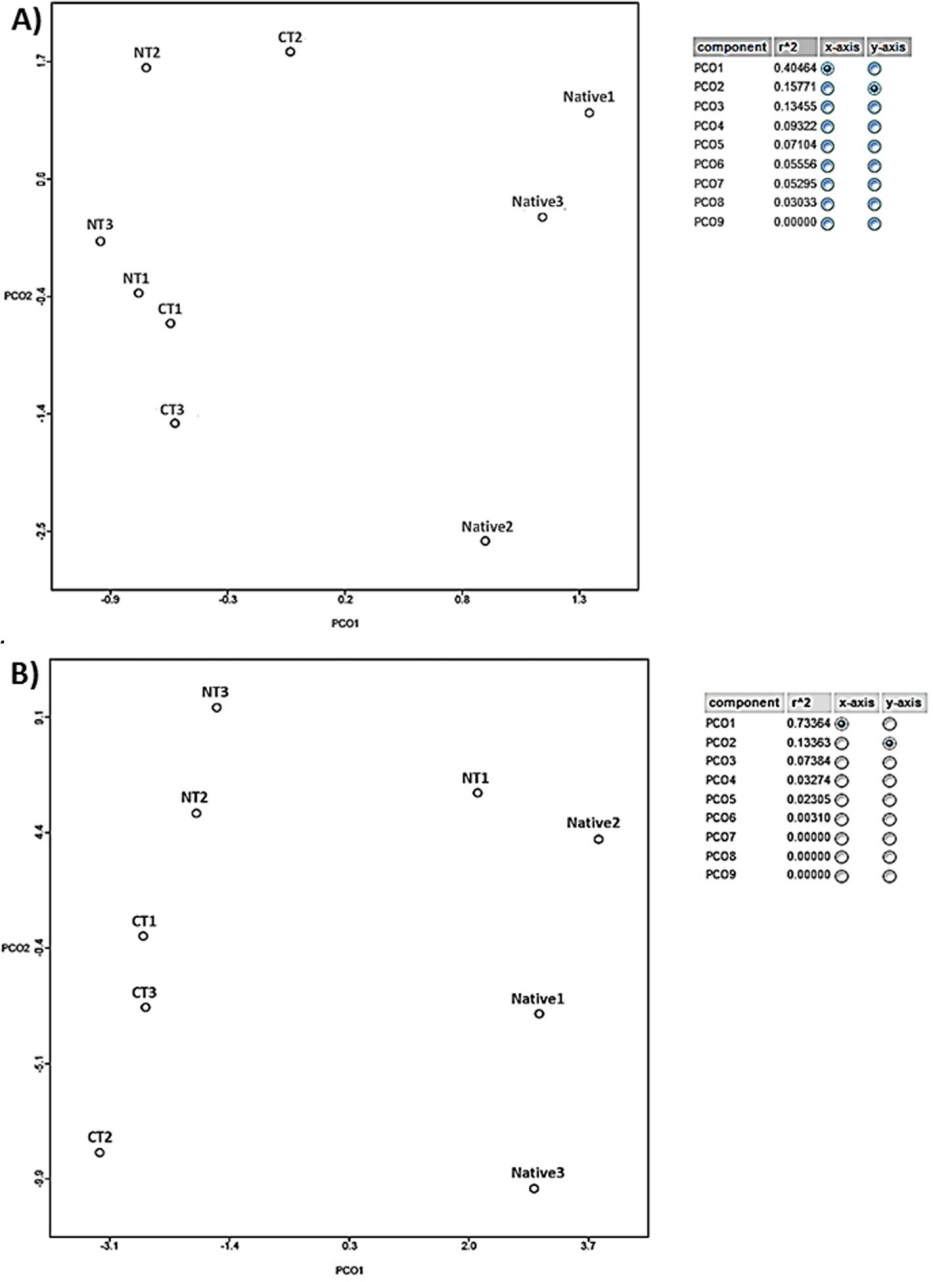
PCoA analysis generated in (**a**) MG-RAST abundance compared to M5NR database and (**b**) of functional categories of subsystems generated in MG-RAST using normalized values (between 0 and 1) and Bray-Curtis distance for no-tillage (NT), conventional tillage (CT) and undisturbed Cerrado (Native) soil metagenomes

The results obtained in the PCoA analysis indicated that agricultural and undisturbed Cerrado soils had different taxonomic profiles (Fig. 1a), and similar results were observed for the functional profiles (Fig. 1b).

### Microbial community composition

**Fig. 2:**
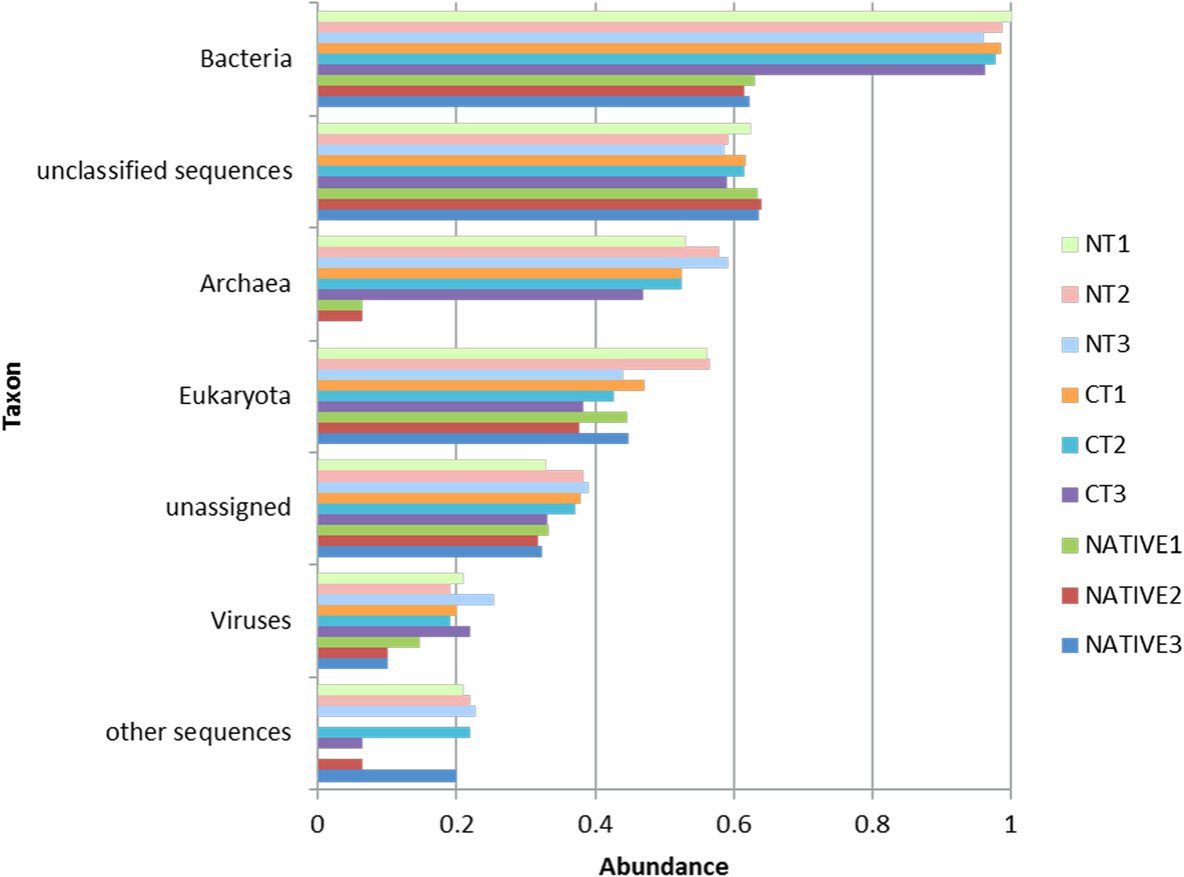
Sequence abundance at the Domain level compared to M5NR database using normalized values between 0 and 1 for no-tillage (NT), conventional tillage (CT) and undisturbed Cerrado (Native) soil metagenomes. For Bacteria, Archaea and Viruses, *p* < 0.05 in the comparison of NT or CT with the Cerrado, but not between NT and CT

The community structure analyses performed with the M5NR (M5 non-redundant protein) database available in the MG-RAST server [20] showed that in all treatments the majority of the sequences were attributed to the Bacteria domain, and the remaining were unclassified sequences (sequences that do not fit into the established parameters of size of sequences) of Archaea, Eukaryota, unassigned (unknown sequences showing no similarity with any known sequences), Viruses and other sequences (including other sequences as small RNAs or regulation motifs) (Fig. 2).

Differences in microbial composition at the domain level were detected among the treatments. The largest was observed in Bacteria domain, where the majority of the sequences were assigned to the NT and CT treatments, while the undisturbed soil had considerable lower numbers of sequences (*p* < 0.05 in the comparison of NT or CT with the Cerrado, but not between NT and CT). The second largest domain was of unclassified sequences equally distributed in all treatments. Interestingly, Archaea were very low in two replicates of the native Cerrado, but very abundant with the introduction of agriculture (*p* < 0.05). Eukarya was higher in two replicates of the NT treatment, but with no statistical difference between the treatments, and Viruses were lower in the native soil (*p* < 0.05) (Fig. 2).

**Fig. 3:**
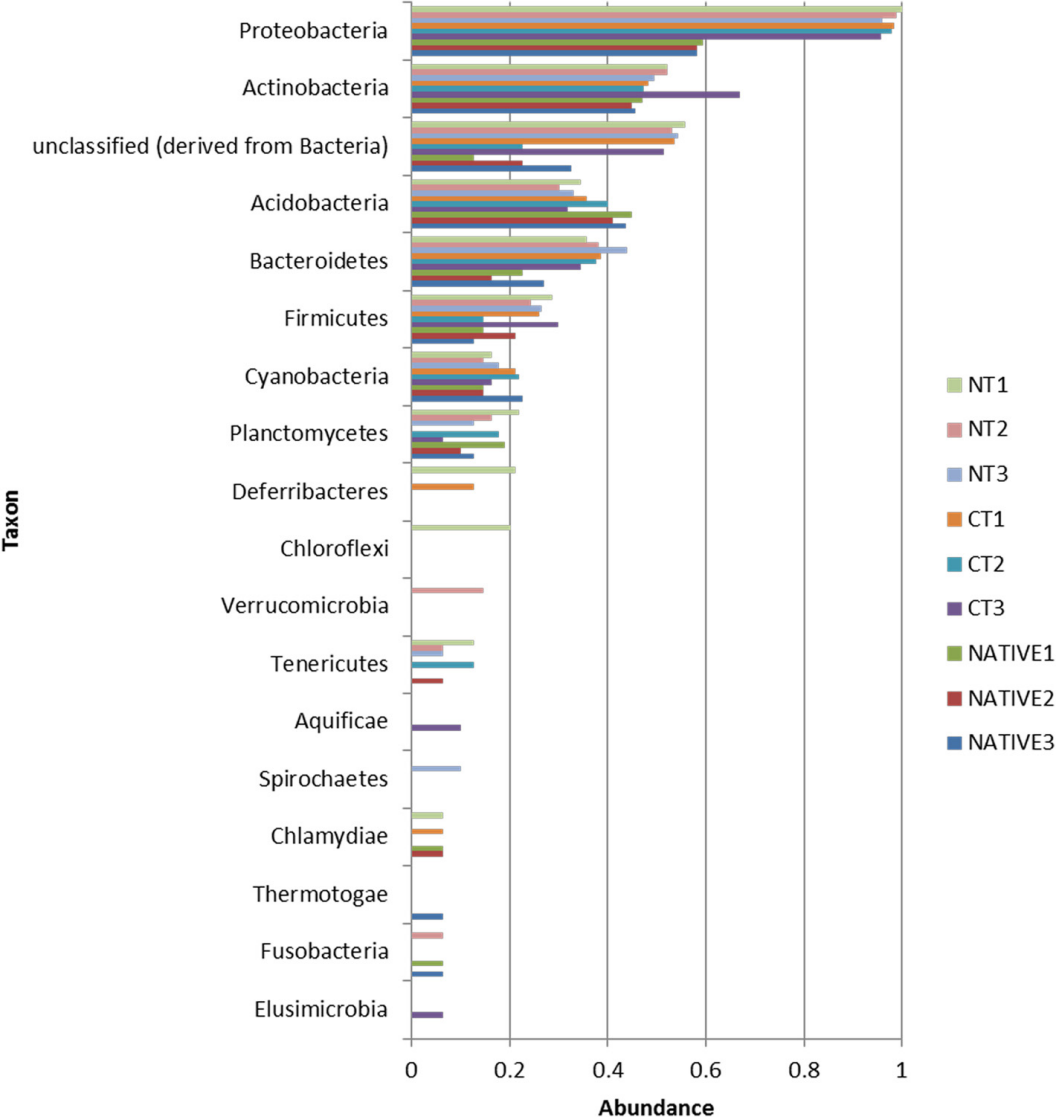
Sequence abundance phylum of Bacteria Domain compared to M5NR database using normalized values between 0 and 1 for no-tillage (NT), conventional tillage (CT) and undisturbed Cerrado (Native) soil metagenomes. For the Proteobacteria, Unclassified, Bacteroidetes and Firmicutes, *p* < 0.05 in the comparison of NT or CT with the Cerrado, but not between NT and CT; while for the Acidobacteria, the Cerrado was significantly higher, *p* < 0.05

Among the Bacteria, the most abundant phylum was Proteobacteria in both NT and CT, in comparison to the native area (*p* < 0.05) (Fig. 3). The two most abundant classes of Proteobacteria were Alphaproteobacteria and Betaproteobacteria (data not shown). Actinobacteria was the second most abundant phylum of the Bacteria domain, and in general was not very different among the treatments, except for one replicate of the CT. The Bacteroidetes, Firmicutes and unclassified sequences derived from Bacteria were more abundant in NT and CT treatments in comparison to the Cerrado, while the Acidobacteria phylum dominated in the native soil (*p* < 0.05) (Fig. 3).

**Fig. 4:**
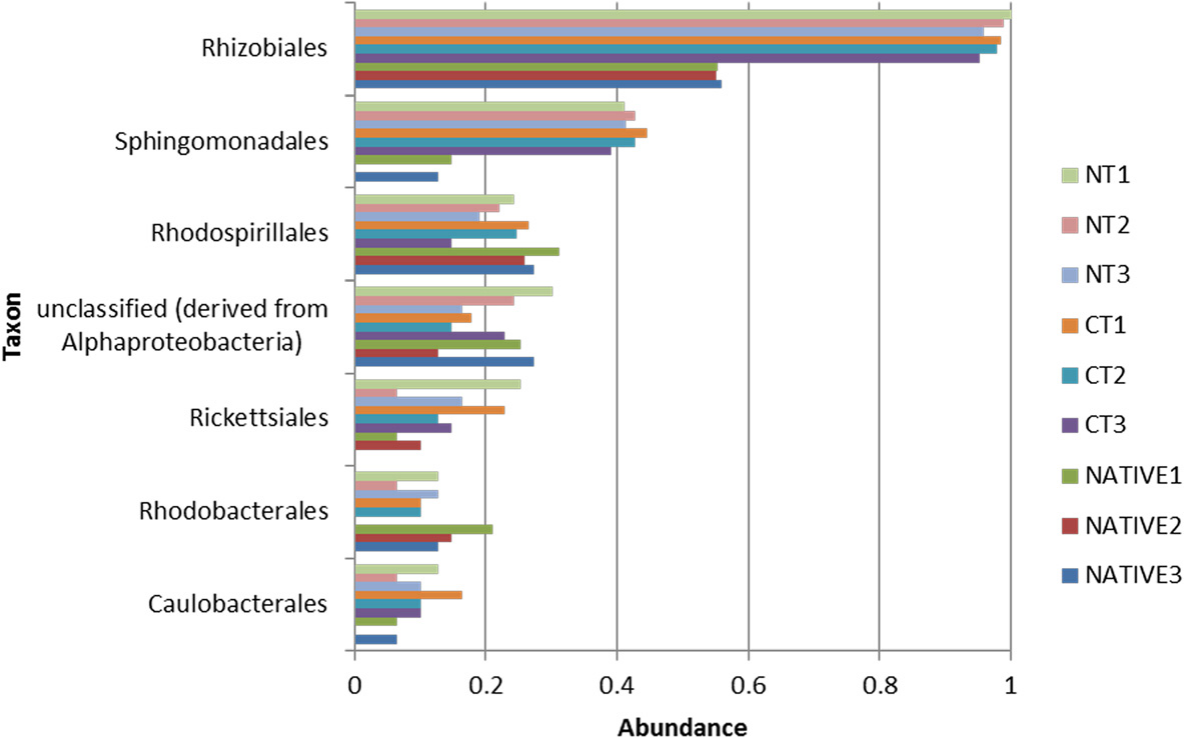
Sequence abundance orders of Alphaproteobacteria compared to M5NR database using normalized values between 0 and 1 for no-tillage (NT), conventional tillage (CT) and undisturbed Cerrado (Native) soil metagenomes. For the Rhizobiales and Sphingomonadales, *p* < 0.05 in the comparison of NT or CT with the Cerrado, but not between NT and CT

**Fig. 5:**
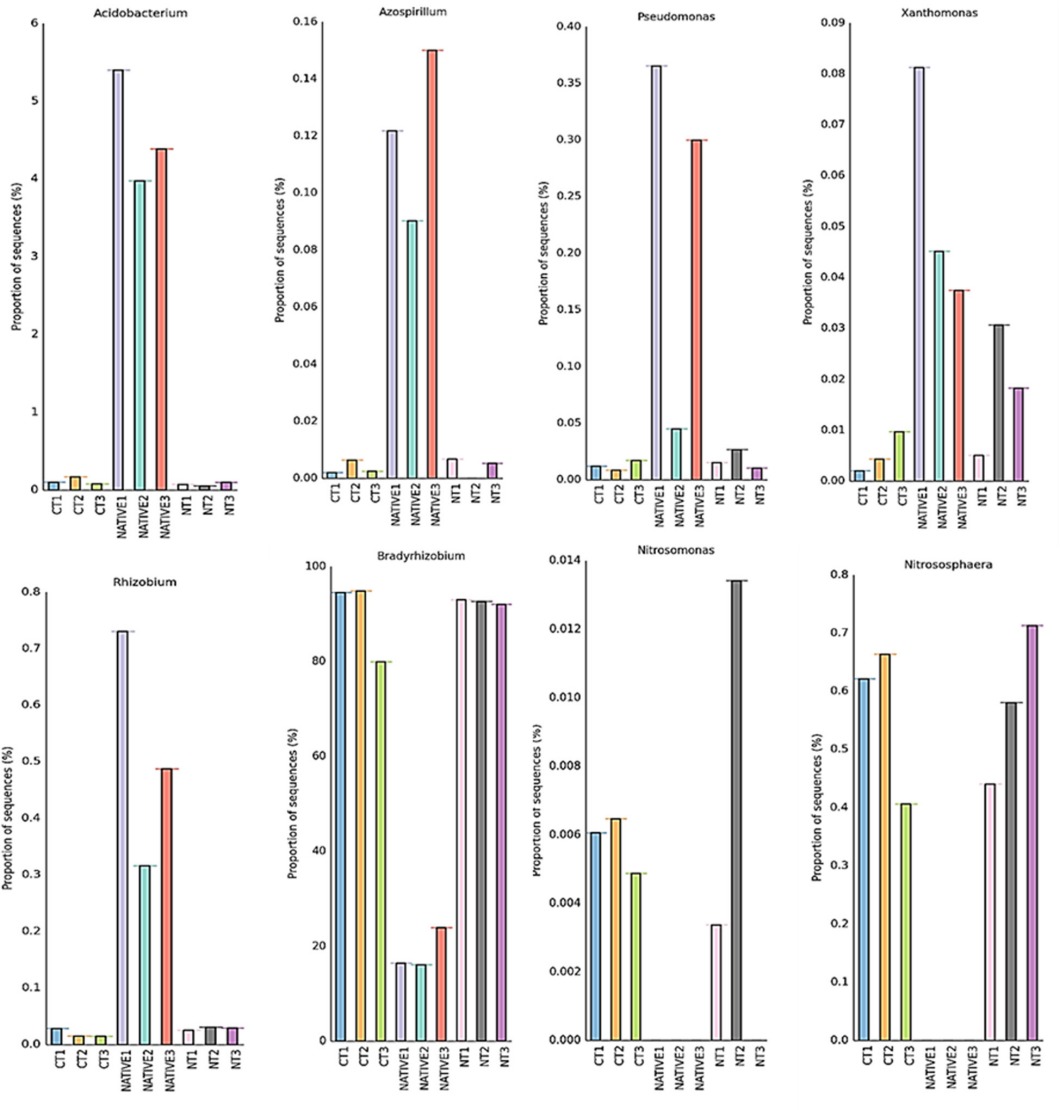
Proportion of sequences of the main genera generated in STAMP software using MG-RAST genus abundance profiles (M5NR database) for no-tillage (NT), conventional tillage (CT) and undisturbed Cerrado (Native) soil metagenomes. *Bradyrhizobium*, *Nitrosomona*s and *Nitrosphaera* were significantly higher in the CT and NT in comparison with the Cerrado, while the others were higher in the Cerrado

We will focus on the results that have shown statistical differences between the treatments. The order Rhizobiales was the most dominant in Alphaproteobacteria in the CT and NT metagenomes (*p* < 0.05) (Fig. 4). Within the Rhizobiales, although the genus *Rhizobium* was significantly higher in native soils, *Bradyrhizobium* was higher in the soils under CT and NT (Fig. 5). Still in the Alphaproteobacteria, the genus *Azospirillum* was more abundant in the undisturbed soil (*p* < 0.05) (Fig. 5). Within the Alphaproteobacteria, the Sphingomonadales was also higher in the CT and NT systems (*p* < 0.05) (Fig. 4).

In the Betaproteobacteria class, the order Burkholderiales was the most abundant in the NT system, followed by Nitrosomonadales, both in the CT and NT systems (*p* < 0.05) (Additional file 2: Figure S2). In the Betaproteobacteria, the genus *Nitrosomonas* was more abundant in the cropped areas, while in the Gamaproteobacteria the *Pseudomonas* and *Xanthomonas* were significantly more abundant in the undisturbed soil (*p* < 0.05) (Fig. 5). The genus *Acidobacterium* of the phylum Acidobacteria was also higher in undisturbed soil (*p* < 0.05) (Fig. 5).

Within the Archaea domain, the Crenarchaeota phylum was the most abundant in the NT soil, while the Thaumarchaeota phylum was the second most abundant and unclassified Archaea the third, in the NT and CT treatments, and all were practically not detected in the native soil (*p* < 0.05) (Additional file 2: Figure S3). Within this last phylum, the genus *Nitrosphaera* was more abundant in the NT and CT treatments (Fig. 5).

As pointed out before, the Eukaryota domain was more abundant in the NT treatment (Fig. 2), where there was dominance of the phylum Ascomycota (*p* < 0.05), followed by unclassified sequences and of Streptophyta, but these without statistical difference (Additional file 2: Figure S4). The low number of Viruses sequences, dominant in the CT and NT treatments (Fig. 2), was represented only by the Caudovirales order, higher in the CT and NT treatments in comparison with the Cerrado (*p* < 0.05) (Additional file 2: Figure S5).

### Functional metagenome profiles

**Fig. 6:**
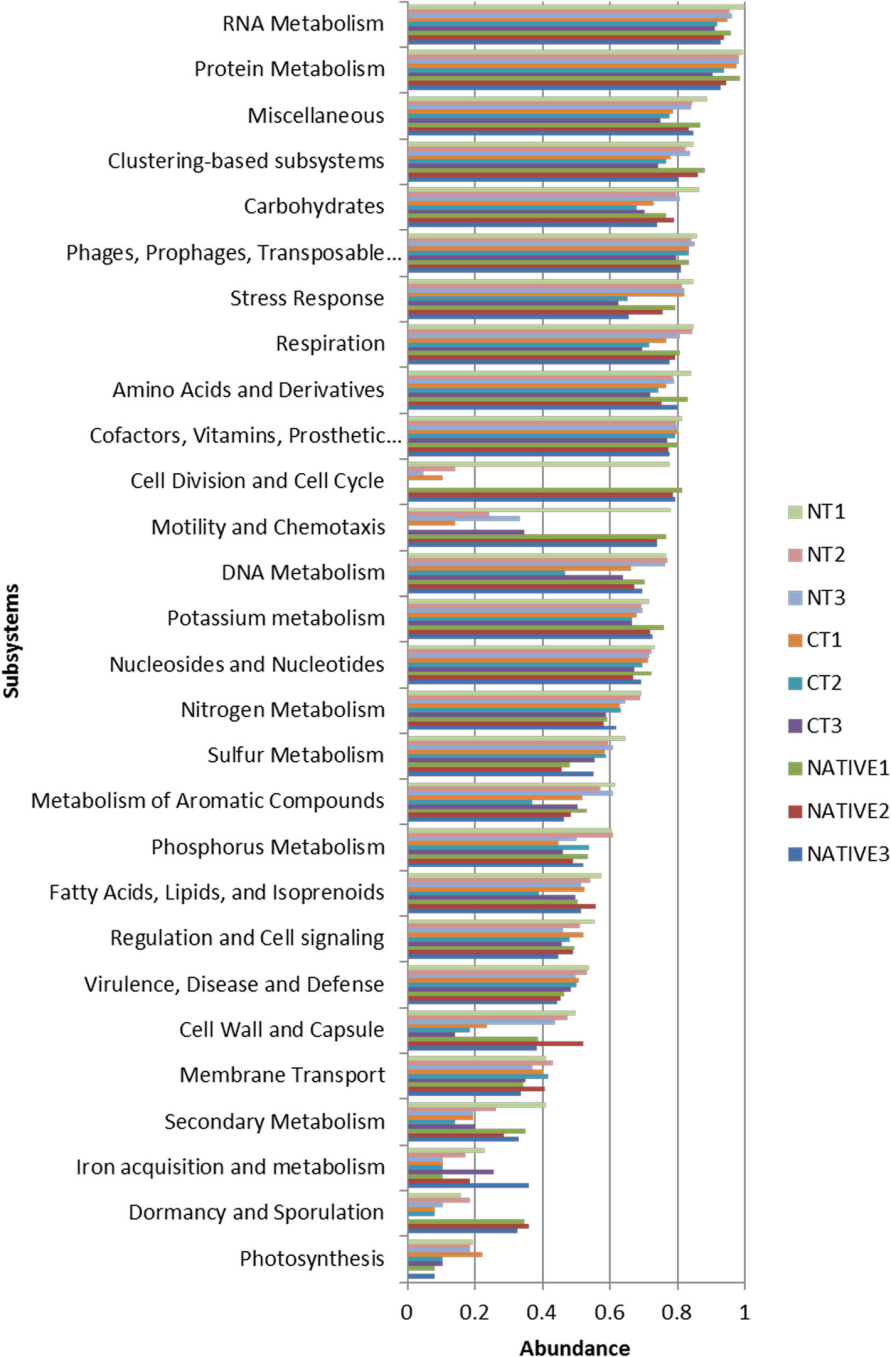
Abundance of functional classification in subsystems categories using normalized values between 0 and 1 for no-tillage (NT), conventional tillage (CT) and undisturbed Cerrado (Native) soil metagenomes

Functional analysis generated by MG-RAST classified the sequences in 29 subsystems (Fig. 6), based on the relative abundance of the data normalized on a scale from 0 to 1. The five categories with more sequences were the RNA metabolism, protein metabolism, miscellaneous, clustering-based subsystems (functional coupling evidence but unknown function) and carbohydrates. The NT and the native soil showed similar numbers of sequences in all these subsystems, while the CT showed lower numbers of sequences. The CT had also lower numbers of sequences in other categories, including stress response, respiration, amino acids and derivatives, cell division and cell cycle. For the undisturbed area, we can mention higher numbers in the subsystems of cell division and cell cycling, motility and chemotaxis, dormancy and sporulation (Fig. 6).

## Discussion

### General Characterization of the Cerrado soils before and after the introduction of agricultural practices

The Brazilian Cerrado currently represents the most important grain producing area in the country, besides covering 24 % of the Brazilian land [1]. The edaphoclimatic conditions of the Cerrado find some parallel with the African savannahs, and in both cases, there are still few studies about microbial communities. The typical soil chemical properties of the Cerrado are of high Al content, low pH and low P, such that the use of lime and fertilizers is necessary to allow economic crop production and results in increased level of soil nutrients (Table 2). The no-tillage (NT) system is being increasingly adopted in the Cerrado over the conventional tillage (CT). Reduced soil disruption and soil cover by plant residues in the NT result in improved physical and chemical properties over the CT, including higher organic matter (OM) content, improved water retention capacity and lower oscillation of temperatures (e.g. [21–24]). Our results confirmed higher OM and nutrient contents (Ca, K, Mg, P) in the NT in comparison to the CT after 23 years of cropping (Table 2).

As observed before [25, 26], significant reductions in microbial biomass (MB-C, MB-N), acid phosphatase and arylsulfatase activities were observed in the CT areas in comparison to the NT and the native Cerrado (Table 3). Reduction in activity of both enzymes should be related to both the reduction in OM and the addition of chemical fertilizers when agriculture was established in the area. Contrarily, β-glucosidase (BG) activity was higher in the NT than in the Cerrado and in the CT, possibly due to the quality and quantity of plant residues, which are more complex in the undisturbed Cerrado and in the CT, since the β-glucosidase acts in less complex residues [25–27].

### Microbial taxonomic and functional diversity

The shotgun approach in metagenomic studies allows better understanding about soil microbial communities, indicating not only the taxonomic groups, but also metabolic functions. The approach has already been successful in detecting differences in the composition and functionality of microbial communities in the comparison of NT and CT in a fertile oxisol of southern Brazil, subtropical climate [15, 19]. Now, in a different edaphoclimatic condition and having an undisturbed area for comparison, we confirmed that both soil managements caused profound changes in microbial structure and functioning (Fig. 1a, b).

In general higher taxonomic diversity was not associated to the native Cerrado, but rather to agricultural soils which showed higher abundances of Bacteria, the predominant domain in the soil, as well as of Archaea and Viruses (Fig. 2), in agreement with other studies carried out in Brazil [28], North America [29] and Europe [30]. The results indicate that the stresses imposed by agriculture modify soil microbiome by increasing its taxonomic diversity. For example, also using shotgun analyses Mendes et al. [31] have shown that the soybean rhizosphere selected taxonomic and functional communities for its best development. Shifts may also be necessary to support the newly disrupted environment.

As in other metagenomic studies [15, 32, 33], Proteobacteria were dominant in all soils; however, one interesting observation of our study was the increase of this phylum in the NT and CT treatments (Fig. 3), and emphasis should be given to the genus *Bradyrhizobium* (Fig. 5). Brazilian soils are free of *Bradyrhizobium* compatible with soybean [11], and massive inoculation is usually practiced every cropping season [34]. Genetic events such as high rates of horizontal transfer of symbiotic genes from the inoculant to indigenous rhizobia have been reported in the Cerrado as a result of massive inoculation [35, 36], but no negative impacts on yield have ever been reported [34]. Now we show that massive inoculation can indeed affect soil microbial communities, and soil enrichment with inoculant strains might help to explain some failures in introducing new strains in soils with established populations [34, 37]. Interesting, *Bradyrhizobium* is related to the nitrogen metabolism subsystem, also more abundant in the agricultural soils (Fig. 6). In contrast, in the undisturbed Cerrado there was higher abundance of *Rhizobium* (Fig. 5), although in a proportion 100-times lower than *Bradyrhizobium* in cropped soils (Fig. 5). Interestingly, studies with classical methods performed in undisturbed Cerrado areas have reported high abundance of *Rhizobium* species tolerant of acidity and stressful environmental conditions (e.g. [38]), indicating adaptation to the typical edaphoclimatic conditions of the region.

Other groups that are critical for soil functioning and more abundant in agricultural soils were Burkholderales and Nitrosomonadales (Additional file 2: Figure S2). The *Burkholderia* are highly versatile in their ecological niches, including agricultural soils [39], where they play important roles in soil bioremediation [10], plant growth promotion and biological nitrogen fixation [8, 40, 41]. Nitrogen-fixing *Burkholderia* are abundantly found in the Cerrado, especially in symbiosis with *Mimosa* spp., plants that have this biome as their major center of diversity [42]; in addition, *Burkholderia* can colonize diverse host plants [43]. Nitrosomonadales are related to nitrification processes [44], fitting into the nitrogen metabolism subsystem (Fig. 6), and their superiority in cropped soils (Additional file 2: Figure S2) may reflect the use of N-fertilizer inputs to the maize crop, or N residues left by the soybean crop.

Acidobacteria plays several functions in soils, including the degradation of polymers and soil contaminants [45, 46], and the Cerrado is well known for the richness in these microorganisms [1, 28, 47–50]. This group of microorganisms was more abundant in the Cerrado (Fig. 3), in agreement with previous comparisons between undisturbed Cerrado and areas with agriculture and pastures. In general, Actinobacteria were found in similar abundances in all three treatments (Fig. 3). Reports about Actinobacteria vary with the biome; in Amazon the phylum was higher in undisturbed than in deforested soil [51], while in Cerrado converted to pasture was higher than in the native area [52]. *Streptomyces* is the most common Actinobacteria genus in nature, and predominantly found in soils [53, 54]. The genus has high ability to synthesize metabolites such as antibiotics [55, 56], and several studies in the Cerrado biome have reported copious presence of these microorganisms [50, 57]. Their biotechnological importance as promising sources of chitinases, proteases and xylanases [58–60] should also be mentioned. In addition, their antagonism against several microorganisms can represent a useful biological control tool [60, 61], and they may also interfere with the introduction of beneficial microorganisms, as reported for inoculant strains of *Bradyrhizobium* [62].

Other microorganisms such as Bacteroidetes and Firmicutes were also abundant in our study, especially in agricultural soils (Fig. 3). The Firmicutes phylum includes the classes *Bacilli* and *Clostridia* that are well known spore-forming microorganisms, resulting in greater chance of survival in disturbed environments. There are also reports that these microorganisms are dominant in environments rich in P [63], and in our study the input of P-fertilizer to the cropped soils raised considerable their P content. In addition, this might explain the increase of sequences in the P metabolism subsystem in the NT treatment (Fig. 6). Bacteroidetes are usually very common in soils [64] and in one study were more abundant in agricultural ecosystems, in comparison to a forest soil [63]. Nacke et al. [30] observed that the relative abundances of Bacteroidetes increased with higher pH values, in agreement with the results from our study.

It has been suggested that soil pH [65, 66] and plant residues [67–69] greatly affect the diversity and activity of soil microbial communities. Regarding soil pH, a good example is Acidobacteria, very abundant in the acidic native Cerrado soils [1, 48, 70] and also found in our study. However, every biome has different responses to soil disturbance. For example, in our study the abundant groups Acidobacteria and Alphaproteobacteria were affected by agriculture introduction, with decreases and increases in taxonomic diversity, respectively. In contrast, Navarrete at al. [51] found no differences in the same groups when compared an Amazon forest soil and a soil under slash-and-burn clearing.

As reported before in other metagenomic studies [71], there were few sequences of Archaea, and they were practically absent in the native Cerrado (Fig. 2). In a previous study, we detected more Archaea in the NT than in the CT system in southern Brazil [15], and the result was attributed to a negative impact of tillage on this domain. Similar results were now confirmed in the NT vs. CT soils of the Cerrado, but they were— as also observed in another Cerrado soil [70]—surprisingly low in the undisturbed soil. However, we must consider the observations of Rodrigues et al. [72], showing increase in richness and diversity of Archaea in the dry season, and our samples were obtained in the rainy season. Crenarchaeota, the most abundant phylum, was present only in the NT and Thaumarchaeota, the second most abundant, was present in both NT and CT systems (Additional file 2: Figure S3); both phyla are relevant in agricultural soils due to their role in the nitrification process [72–74].

Eukaryota was the third most abundant domain, with higher number of sequences in the NT treatment (Fig. 2), and predominance of the Ascomycota phylum (Additional file 2: Figure S4). These results are in agreement with Castro et al. [75], that reported that human activity increased this phylum in comparison to the native soil, what could be related to a higher tolerance to environmental stresses [15, 76]. The phylum includes a variety species that go from plant pathogens to decomposers of organic matter, but they are also important in undisturbed areas [77]. Disking in the CT system would favor hyphae disruption, decreasing fungi population [78], but the subject needs more studies to be clarified, as in southern Brazil Eukaryota were more abundant in the CT, with a possible explanation relying on the higher tolerance of fungi to environmental stresses [15].

Although Viruses sequences were low (Fig. 2), Caudovirales was higher in agricultural soils (Additional file 2: Figure S5). The order consists of bacteriophages commonly found in soils, and their role in infecting Archaea and Bacteria may help in population control [79]; moreover, [80] showed that this predation is important for the control and growth promotion of bacterial population in soil.

In relation to microbial functions, the two most abundant subsystems, RNA and protein metabolism (transcription, translation, protein folding and degradation) are attributed to constitutive genes (Fig. 6). Miscellaneous (e.g. iron-sulfur cluster assembly and histidine degradation) was the third more abundant subsystem in the NT, in agreement with other studies, where they are usually positioned among the four most abundant subsystems [81, 82]. Classification of sequences as “clustering-based” indicates an unknown function, and in general, this represents the most abundant subsystem in soil metagenomes [19, 81, 83]. In our study, these subsystems included genes such as cytochrome biogenesis, proteases, cell-cycling and cell division and were higher in the undisturbed soil, followed by the NT. The carbohydrates subsystems, including central carbohydrate metabolism and fermentation, were more abundant in the NT, what could be related to the soil enrichment with crop residues [21, 23, 24]. The increased β-glucosidase activity levels under NT, for instance, are closely associated with the composition of plant residues [27, 84].

## Conclusions

Our study highlights that the Brazilian Cerrado soils encompass high taxonomic and functional diversity of soil microorganisms; however, both are highly impacted by agriculture. Interestingly, as pointed out by Fierer et al. [71], functional diversity was not necessarily associated with the taxonomic diversity, as the least conservation system, the CT treatment, presented increased taxonomic sequences and reduced functional metagenomic profiles in comparison to the undisturbed Cerrado. That might indicate a strategy in the CT to try to maintain soil functioning by favoring taxa that are probably not the most efficient for some functions, leading to negative impacts in soil quality with time. In addition, in general agricultural soils changed to be more adapted to degrade accessible carbon and aromatic compounds substrates, as well as to be enriched in microorganisms related to the metabolism of N, P and S, as a response to the addition of fertilizers. We should also mention that native soils were rich in unknown functions, emphasizing the possibility of finding new functions and genes.

The typical vegetation of Cerrado, represented by stress-tolerant plant species, adapted to harsh conditions such as highly-weathered acidic soils, poor in nutrients, high temperatures, natural fire and long dry periods, might let us think that the biome could be less affected by anthropogenic activities. With the help of a metagenomic approach we have obtained new results that confirm previous observations using classical methods [13], showing that the Cerrado is, together with the Amazon, the most fragile biomes in Brazil. Underneath the rustic appearance of the Cerrado vegetation there is a fragile soil microbial community.

## Methods

### General description of the areas and soil sampling

Soil samples were collected at the experimental station of Embrapa Cerrados in Planaltina, Federal District, Brazil (15°36′34” S and 47°44′36” W). The altitude of the sites is approximately 1170 m, the climate is tropical seasonal (Aw, Köppen classification), with average rainfall of 1500 mm concentrated in the period from September to April, and a dry period lasting 5–6 months. The average annual temperature is 21 °C, with an average high of 28 °C in September and an average low of 17 °C in June. The soil is classified as Latossolo Vermelho Amarelo argiloso (Brazilian system), clayey Typic Haplustox (US classification). The area relief is mostly plan.

The treatments were initially established in a very homogenous area. The area was transformed in two large experiments with two soil management systems. At the time of our study the experiments were 23-year-old under conventional tillage (CT) or no-tillage (NT), both cropped with soybean (*Glycine max* (L.) Merr.) in one rainy season and maize (*Zea mays* L.) in the following one, and left as fallow in the winter (dry season). The CT area was annually prepared by ploughing and disking the soil before sowing and for incorporation of weeds after harvest, whereas the NT area was managed without ploughing or disking. Plots of CT measured 25 m width x 320 m length and plots of NT measured 50 m width x 320 m length. A treatment representing the undisturbed Cerrado *stricto sensu* (native) was included as a reference for the original soil conditions. The area has no history of anthropogenic activity and represents a typical area of native Cerrado *stricto sensu.*

Soils were sampled from each area in January of 2014 during the rainy season (summer), at 0–10 cm depth. Each sampling area under NT (8000 m^2^), CT (11200 m^2^), and native Cerrado (6700 m^2^) was split into three sub-areas in order to generate three replicates. Therefore, each biological replicated corresponded to 2267 m^2^, 3733 m^2^ and 2233 m^2^ for the NT, CT and native Cerrado, respectively. From each sub-area of each treatment ten subsamples spatially distributed to cover the whole area were taken to form a composite sample. Therefore, each of the three treatments ended up with three replicates, each composed by ten subsamples. At the sampling time the CT and NT area had maize at flowering stage.

Soil samples were placed in plastics bags and transported to the laboratory, plant residues and roots were removed and soil was sieved (<4 mm, 5 mesh). Subsamples were sent to chemical and physical analyses; others were stored at 4 °C for microbial biomass and soil enzymes analyses, the remaining being kept at −20 ° C for the metagenomic analysis.

For chemical and physical analyzes, samples were air-dried and sieved again through a 2-mm mesh for chemical analyses using routine methods [85]. Soil pH was measured at a soil:water ratio of 1:2.5 by weight. Ca, Mg and Al were extracted with 1 N KCl and quantified through atomic absorption (Ca and Mg) and titration with NaOH 0.025 M (Al); P and K were extracted using the Mehlich 1 (H_2_SO_4_ 0.0125 M + HCl 0.05 M) method, and quantified through flame spectrophotometry (K), or by using the blue-Mo method (P). Soil organic matter (SOM) was determined using the Walkley and Black method. Soil physical properties were analyzed using routine methods [85].

### Microbial biomass and enzymes activity

To characterize and compare biological activity we evaluated microbial biomass-C and –N (MB-C, MB-N), and the soil enzymes β-glucosidase, arylsulfatase and acid phosphatase. Analyses were performed in each of the three replicates, each with three analytical replicates.

The soil MB-C and MB-N were determined using the chloroform-fumigation-extraction method [78]. C and N in the fumigated and non-fumigated samples were determined using a total organic C and N analyzer (Vario TOC Cube, Elementar Analyser System GmbH) with an infrared detector. For the calculation of MB-C and MB-N, k_CE_ and k_NE_ factors of 0.35 [86] and 0.54 [87] were used.

The β-glucosidase (E.C. 3.2.1.21), acid phosphatase (E.C. 3.1.3.2), and arylsulfatase (E.C.3.1.6.1) activities were determined according to M Tabatabai [88]. Due to their short incubation periods (1 h), toluene was omitted from the assays. These three soil enzymes were selected for their roles in the C cycle (β-glucosidase), P cycle (acid phosphatase), and S cycle (arylsulfatase), respectively.

### Metagenome

#### DNA extraction and shotgun sequencing

Metagenomic DNA was extracted using the PowerMax™ Soil DNA Isolation Kit (MoBio Laboratories), following the manufacturer’s procedure, and submitted to sequencing analysis in the Ion Torrent PGM sequencing platform (Life Technologies) at the Bioinformatics Laboratory of LNCC Petrópolis, Rio de Janeiro, Brazil, (http://www.lncc.br). Nine libraries of 400-base-pairs DNA fragments, using 100 ng of DNA from each sample were constructed. The libraries were prepared according to Ion Xpress™ Plus gDNA Fragment Library Preparation protocol. For DNA fragmentation the Ion Shear™ Plus Reagents were used. Emulsion PCR was carried out in the Ion OneTouch™ 2 System. Each library was unidirectionally sequenced in one Ion 318™ Chip v2 using an Ion PGM™ System. The metagenomic fragments were submitted to FastX-trimmer (http://hannonlab.cshl.edu/fastx_toolkit/) in order to remove low quality sequences (phred score < 15) and short reads (<=50 bp). The duplicated reads were filtered using the Replicates software [89]. The retained sequences were submitted to MG-RAST v.3.3 server [20].

#### Taxonomic and functional analyses

The sequenced fragments from the nine metagenomes were deposited on MG-RAST v.3.3 (the Metagenomics RAST – http://metagenomics.anl.gov) with the accession numbers 4577670.3 (CT_1), 4578926.3 (CT_2), 4578927.3 (CT_3), 7577671.3 (NT_1), 4578714.3 (NT_2), 4577672.3 (NT_3), 4577669.3 (NATIVE_1), 4578924.3 (NATIVE_2), and 4578925.3 (NATIVE_3). For the taxonomic classification, the sequences were compared against M5NR (M5 non-redundant) database based on the “best hit classification” method. The rarefaction curve and Principal Coordinates Analysis (PcoA) were derived from MG-RAST, estimated with the table of abundances for comparative analyses. The parameters used were: *Max. e-value Cutoff:* 1e^−5^; *Min. % Identity Cutoff*: 80 %; *Min. Alignment Length Cutoff: 50*. These filters were used to avoid false positive sequences. It is worth mentioning that the classification in MG-RAST includes the categories of unclassified sequences (sequences that do not fit into the established parameters of size of the sequences), the unassigned category (unknown sequences showing no similarity with any known sequences) and the category of other sequences (including other sequences as small RNAs or regulation motifs). The PcoA was performed using the default parameters. For the functional analysis, the sequences were compared against SEED database and classified in subsystems [90] using the hierarchical classification method based on the distance method of Bray-Curtis.

### Statistical analyses

For chemical properties, MB-C and MB–N and soil enzymes data were analyzed by one-way analysis of variance (ANOVA). Statistical differences between means were assessed by Tukey’s test (p <0.05). All assumptions required by the analysis of variance were verified. These analyses were performed in MSTAT-C (Michigan State University)

To facilitate comparative analyses, visualization and statistical tests of abundance, the metagenome data were normalized with a log transformation, and this procedure is applied to each distribution in a group of distributions so that all distributions exhibit the same mean and the same standard deviation. Thereby all values are placed on a scale from 0 to 1, showing all abundance counts in a more intuitive scale [91]. For the metagenome data, the abundance profiles obtained from MG-RAST were submitted to STAMP (Statistical Analysis of Metabolic Profile) software [92], to identify genus and functions statistically different among all treatments. As for each treatment three replicates were analyzed and data were not pooled, and several combinations of pairs were analyzed by STAMP.

For taxonomic data, the ANOVA test was used (*p <* 0.05), Tukey-Kramer as post-hoc, and Storey’s FDR (false discovery rate) for correction. First, the data were not grouped, but the Storey FDR test indicated that there were no statistical differences within each group. Then the samples from each treatment were pooled. For function analyses, the metagenomes were grouped according the treatments (CT1, CT2, CT3 - as group 1; NATIVE1, NATIVE2, NATIVE3 - as group 2, and NT1, NT2, NT3- as group3) and analyzed using the two groups approach, with Welch’s *t*-test, Welch’s inverted as CI method, and Storey FDR for correction. Each sample replicate was considered on the statistic test. Categories with biological relevance were obtained using a difference of proportions of 1 and ratio of proportions of 2 as filters.

## Additional files

Additional file 1: Table S1. Main features of the compositing metagenomes based on the MG-RAST annotations. Treatments correspond to soils under native vegetation of Cerrado (Native) or cropped with soybean/corn under no-tillage (NT) or conventional tillage (CT) systems. (DOCX 18 kb) Additional file 2: Figure S1. Rarefaction curves generated with the MG-RAST software against M5NR database using normalized values between 0 and 1 for no-tillage (NT), conventional tillage (CT) and undisturbed Cerrado (Native) soil metagenomes. **Figure S2.** Sequence abundance orders of Betaproteobacteria compared to M5NR database using normalized values between 0 and 1 for no-tillage (NT), conventional tillage (CT) and undisturbed Cerrado (Native) soil metagenomes. The order Burkholderiales was the most abundant in the NT system, followed by Nitrosomonadales, both in CT and NT (*p* < 0.05). **Figure S3.** Sequence abundance of phyla of Archaea Domain compared to M5NR database, and using normalized values between 0 and 1 for no-tillage (NT), conventional tillage (CT) and undisturbed Cerrado (Native) soil metagenomes. Crenarchaeota was higher in the NT, while Thaumarchaeota and unclassified were higher in the NT and CT treatments (*p* < 0.05). **Figure S4.** Sequence abundance of the phyla of Eukaryota Domain compared to M5NR database and using normalized values between 0 and 1 for no-tillage (NT), conventional tillage (CT) and undisturbed Cerrado (Native) soil metagenomes. **Figure S5.** Sequence abundance in the Viruses domain compared to M5NR database using normalized values between 0 and 1 for no-tillage (NT), conventional tillage (CT) and undisturbed (Native) soil metagenomes. Caudovirales was higher in the NT and CT systems (*p* < 0.05). (DOCX 423 kb)

## References

[1] Araujo J, de Castro A, Costa MC, Togawa R, Júnior GP, Quirino B, Bustamante MC, Williamson L, Handelsman J, Krüger R (2012). Characterization of soil bacterial assemblies in Brazilian savanna-like vegetation reveals acidobacteria dominance. Microb Ecol.

[2] Ruggiero PGC, Batalha MA, Pivello VR, Meirelles ST (2002). Soil-vegetation relationships in cerrado (Brazilian savanna) and semideciduous forest. Southeastern Brazil Plant Ecol.

[3] Viana LT, Bustamante MMC, Molina M, Pinto AS, Kisselle K, Zepp R, Burke RA (2011). Microbial communities in Cerrado soils under native vegetation subjected to prescribed fire and under pasture. Pesq Agropec Bras.

[4] Vogel TM, Simonet P, Jansson JK, Hirsch PR, Tiedje JM, van Elsas JD, Bailey MJ, Nalin R, Philippot L (2009). TerraGenome: a consortium for the sequencing of a soil metagenome. Nat Rev Micro.

[5] Hungria M, Franchini JC, Campo RJ, Graham PH, Werner D, Newton W (2005). The importance of nitrogen fixation to soybean cropping in South America. Nitrogen Fixation in Agriculture, Forestry, Ecology, and the Environment, 4.

[6] Mendes R, Kruijt M, de Bruijn F, Dekkers E, van der Voort M, Schneider JHM, Piceno YM, DeSantis TZ, Andersen GL, Bakker PAHM (2011). Deciphering the rhizosphere microbiome for disease-suppressive bacteria. Science.

[7] Schmidt MWI, Torn MS, Abiven S, Dittmar T, Guggenberger G, Janssens IA, Kleber M, Kogel-Knabner I, Lehmann J, Manning DAC (2011). Persistence of soil organic matter as an ecosystem property. Nature.

[8] Bhattacharyya PN, Jha DK (2012). Plant growth-promoting rhizobacteria (PGPR): emergence in agriculture. World J Microbiol Biotechnol.

[9] Brussaard L. Ecosystem services provided by the soil biota. Soil Ecol Ecosystem Serv. 2012;45–58.

[10] Ali S, Yu F-B, Li L-T, Li X-H, Gu L-F, Jiang J-D, Li S-P (2012). Studies revealing bioremediation potential of the strain *Burkholderia* sp. GB-01 for abamectin contaminated soils. World J Microbiol Biotechnol.

[11] Ferreira MC, Hungria M (2002). Recovery of soybean inoculant strains from uncropped soils in Brazil. Field Crops Res.

[12] Kaschuk G, Alberton O, Hungria M (2010). Three decades of soil microbial biomass studies in Brazilian ecosystems: lessons learned about soil quality and indications for improving sustainability. Soil Biol Biochem.

[13] Kaschuk G, Alberton O, Hungria M (2011). Quantifying effects of different agricultural land uses on soil microbial biomass and activity in Brazilian biomes: inferences to improve soil quality. Plant Soil.

[14] Roesch LFW, Fulthorpe RR, Riva A, Casella G, Hadwin AKM, Kent AD, Daroub SH, Camargo FAO, Farmerie WG, Triplett EW (2007). Pyrosequencing enumerates and contrasts soil microbial diversity. ISME J.

[15] Souza RC, Cantão ME, Vasconcelos ATR, Nogueira MA, Hungria M (2013). Soil metagenomics reveals differences under conventional and no-tillage with crop rotation or succession. Appl Soil Ecol.

[16] Schmidt HF, Sakowski EG, Williamson SJ, Polson SW, Wommack K (2014). Shotgun metagenomics indicates novel family A DNA polymerases predominate within marine virioplankton. ISME J.

[17] Bengtsson-Palme J, Boulund F, Fick J, Kristiansson E, Larsson J. Shotgun metagenomics reveals a wide array of antibiotic resistance genes and mobile elements in a polluted lake in India. Front Microbiol. 2014;5. 10.3389/fmicb.2014.00648 PMC4251439 25520706

[18] Singh KM, Reddy B, Patel D, Patel AK, Parmar N, Patel A, Patel JB, Joshi CG (2014). High potential source for biomass degradation enzyme discovery and environmental aspects revealed through metagenomics of Indian buffalo rumen. BioMed Res Int.

[19] Souza RC, Hungria M, Cantão ME, Vasconcelos ATR, Nogueira MA, Vicente VA (2015). Metagenomic analysis reveals microbial functional redundancies and specificities in a soil under different tillage and crop-management regimes. Appl Soil Ecol.

[20] Meyer F, Paarmann D, D’Souza M, Olson R, Glass E, Kubal M, Paczian T, Rodriguez A, Stevens R, Wilke A (2008). The metagenomics RAST server - a public resource for the automatic phylogenetic and functional analysis of metagenomes. BMC Bioinformatics.

[21] Babujia L, Hungria M, Franchini J, Brookes P (2010). Microbial biomass and activity at various soil depths in a Brazilian oxisol after two decades of no-tillage and conventional tillage. Soil Biol Biochem.

[22] Franchini JC, Crispino CC, Souza RA, Torres E, Hungria M (2007). Microbiological parameters as indicators of soil quality under various soil management and crop rotation systems in southern Brazil. Soil Till Res.

[23] Silva AP, Babujia LC, Franchini JC, Souza RA, Hungria M (2010). Microbial biomass under various soil- and crop-management systems in short- and long-term experiments in Brazil. Field Crops Res.

[24] Silva AP, Babujia LC, Franchini JC, Ralisch R, Hungria M, de Guimarães MF (2014). Soil structure and its influence on microbial biomass in different soil and crop management systems. Soil Till Res..

[25] Mendes I, Souza L, Resck D, Gomes A (2003). Biological properties of aggregates from a Cerrado Oxisol under conventional and no-till management systems. Rev Bras Ci Solo.

[26] Peixoto R, Chaer G, Franco N, Junior FR, Mendes I, Rosado A (2010). A decade of land use contributes to changes in the chemistry, biochemistry and bacterial community structures of soils in the Cerrado. Antonie Leeuwenhoek.

[27] Lopes AAC, Sousa DMG, Chaer GM, Reis Junior FB, Goedert WJ, Mendes IC (2013). Interpretation of microbial soil indicators as a function of crop yield and organic carbon. Soil Sci Soc Am J.

[28] Rampelotto PH, de Siqueira FA, Barboza ADM, Roesch LFW (2013). Changes in diversity, abundance, and structure of soil bacterial communities in Brazilian Savanna under different land use systems. Microb Ecol.

[29] Jangid K, Williams MA, Franzluebbers AJ, Sanderlin JS, Reeves JH, Jenkins MB, Endale DM, Coleman DC, Whitman WB (2008). Relative impacts of land-use, management intensity and fertilization upon soil microbial community structure in agricultural systems. Soil Biol Biochem.

[30] Nacke H, Thürmer A, Wollherr A, Will C, Hodac L, Herold N, Schöning I, Schrumpf M, Daniel R (2011). Pyrosequencing-based assessment of bacterial community structure along different management types in German forest and grassland soils. PLoS ONE.

[31] Mendes LW, Kuramae EE, Navarrete AA, van Veen JA, Tsai SM (2014). Taxonomical and functional microbial community selection in soybean rhizosphere. ISME J.

[32] Pacchioni RG, Carvalho FM, Thompson CE, Faustino ALF, Nicolini F, Pereira TS, Silva RCB, Cantão ME, Gerber A, Vasconcelos ATR (2014). Taxonomic and functional profiles of soil samples from Atlantic forest and Caatinga biomes in northeastern Brazil. MicrobiologyOpen.

[33] Xu Z, Hansen MA, Hansen LH, Jacquiod S, Sørensen SJ (2014). Bioinformatic approaches reveal metagenomic characterization of soil microbial community. PLoS ONE.

[34] Hungria M, Mendes I, de Bruijn F (2015). Nitrogen fixation with soybean: the perfect symbiosis?. Biological Nitrogen Fixation.

[35] Barcellos FG, Menna P, da Silva Batista JS, Hungria M. Evidence of horizontal transfer of symbiotic genes from a *Bradyrhizobium japonicum* inoculant strain to indigenous diazotrophs *Sinorhizobium* (*Ensifer*) *fredii* and *Bradyrhizobium elkanii* in a Brazilian Savannah soil. Appl Environ Microbiol. 2007;73(8):2635–43. 10.1128/AEM.01823-06 PMC1855619 17308185

[36] Batista JSS, Hungria M, Barcellos FG, Ferreira MC, Mendes IC (2007). Variability in *Bradyrhizobium japonicum* and *B. elkanii* seven years after introduction of both the exotic microsymbiont and the soybean host in a Cerrados soil. Microb Ecol.

[37] Mendes IC, Hungria M, Vargas MAT (2004). Establishment of *Bradyrhizobium japonicum* and *B. elkanii* strains in a Brazilian Cerrado oxisol. Biol Fertil Soils.

[38] Ribeiro RA, Rogel MA, López-López A, Ormeño-Orrillo E, Barcellos FG, Martínez J, Thompson FL, Martínez-Romero E, Hungria M (2012). Reclassification of *Rhizobium tropici* type A strains as *Rhizobium leucaenae* sp. nov. Int J Syst Evol Microbiol.

[39] Draghi WO, Peeters C, Cnockaert M, Snauwaert C, Wall LG, Zorreguieta A, Vandamme P (2014). *Burkholderia cordobensis* sp. nov., from agricultural soils. Int J Syst Evol Microbiol.

[40] Suárez-Moreno Z, Caballero-Mellado J, Coutinho B, Mendonça-Previato L, James E, Venturi V (2012). Common features of environmental and potentially beneficial plant-associated *Burkholderia*. Microb Ecol.

[41] Bashan Y, De-Bashan L, Prabhu SR, Hernandez J-P (2014). Advances in plant growth-promoting bacterial inoculant technology: formulations and practical perspectives (1998–2013). Plant Soil.

[42] Reis Jr FB, Simon MF, Gross E, Boddey RM, Elliott GN, Neto NE, Loureiro MF, de Queiroz LP, Scotti MR, Chen WM. Nodulation and nitrogen fixation by *Mimosa* spp. in the Cerrado and Caatinga biomes of Brazil. New Phytol. 2010;186(4):934–46. 10.1111/j.1469-8137.2010.03267.x 20456044

[43] Estrada-De Los Santos P, Bustillos-Cristales R, Caballero-Mellado J (2001). *Burkholderia*, a genus rich in plant-associated nitrogen fixers with wide environmental and geographic distribution. Appl Environ Microbiol.

[44] Monteiro M, Séneca J, Magalhães C (2014). The history of aerobic ammonia oxidizers: from the first discoveries to today. J Microbiol.

[45] Větrovský T, Steffen KT, Baldrian P (2014). Potential of cometabolic transformation of polysaccharides and lignin in lignocellulose by soil *Actinobacteria*. PLoS ONE.

[46] Yergeau E, Sanschagrin S, Beaumier D, Greer CW (2012). Metagenomic analysis of the bioremediation of diesel-contaminated Canadian high arctic soils. PLoS ONE.

[47] Silva AP, Babujia LC, Matsumoto LS, Guimarães MF, Hungria M (2013). Bacterial diversity under different tillage and crop rotation systems in an Oxisol of southern Brazil. Open Agriculture J (TOAJ).

[48] Catão ECP, Lopes FAC, Araújo JF, Castro AP, Barreto CC, Bustamante MMC, Quirino BF, Kruger RH (2014). Soil acidobacterial 16S rRNA gene sequences reveal subgroup level differences between savanna-like cerrado and Atlantic forest Brazilian biomes. Int J Microbiol.

[49] Castro VHL, Schroeder LF, Quirino BF, Kruger RH, Barreto CC (2013). Acidobacteria from oligotrophic soil from the Cerrado can grow in a wide range of carbon source concentrations. Can J Microbiol.

[50] Silva MS, Naves Sales A, Teixeira Magalhães-Guedes K, Ribeiro Dias D, Schwan RF (2013). Brazilian Cerrado soil actinobacteria ecology. BioMed Res Int.

[51] Navarrete AA, Tsai SM, Mendes LW, Faust K, Hollander M, Cassman NA, Veen JA, Kuramae EE (2015). Soil microbiome responses to the short-term effects of Amazonian deforestation. Mol Ecol.

[52] Quirino BF, Pappas GJ, Tagliaferro AC, Collevatti RG, Neto EL, da Silva MRSS, Bustamante MMC, Krüger RH (2009). Molecular phylogenetic diversity of bacteria associated with soil of the savanna-like Cerrado vegetation. Microbiol Res.

[53] Sarigullu F, Emel K, Isil U, Omer C (2013). Determination of antibacterial activities of isolated *Streptomyces* strains from soil at Çukurova University in Turkey. J Food Agr Env.

[54] Bontemps C, Toussaint M, Revol P-V, Hotel L, Jeanbille M, Uroz S, Turpault M-P, Blaudez D, Leblond P (2013). Taxonomic and functional diversity of Streptomyces in a forest soil. FEMS Microbiol Lett.

[55] Saravana Kumar P, Duraipandiyan V, Ignacimuthu S (2014). Isolation, screening and partial purification of antimicrobial antibiotics from soil *Streptomyces* sp. SCA 7. Kaohsiung J Med Sci.

[56] Jauri PV, Bakker MG, Salomon CE, Kinkel LL (2013). Subinhibitory antibiotic concentrations mediate nutrient use and competition among soil *Streptomyces*. PLoS ONE.

[57] Pereira JC, Neves MCP, Drozdowicz A (1999). Dinâmica das populações bacterianas em solos de cerrados. Pesq Agropec Bras., Brasília.

[58] Azeredo LI, Castilho L, Leite SF, Coelho RR, Freire DG, Davison B, Lee J, Finkelstein M, McMillan J (2003). Protease production by *Streptomyces* sp. isolated from Brazilian Cerrado Soil. Biotechnology for Fuels and Chemicals.

[59] Nascimento R, Coelho R, Marques S, Alves L, Gırio F, Bon E, Amaral-Collaço M (2002). Production and partial characterisation of xylanase from *Streptomyces* sp. strain AMT-3 isolated from Brazilian cerrado soil. Enzym Microb Technol.

[60] Gomes R, Semedo L, Soares R, Linhares L, Ulhoa C, Alviano C, Coelho R (2001). Purification of a thermostable endochitinase from *Streptomyces* RC1071 isolated from a cerrado soil and its antagonism against phytopathogenic fungi. J Appl Microbiol.

[61] Anitha A, Rebeeth M (2009). In vitro antifungal activity of *Streptomyces* griseus against phytopathogenic fungi of tomato field. Acad J Plant Sci.

[62] Scotti M, Sá N, Vargas M, Dobereiner J (1982). Streptomycin resistance of *Rhizobium* isolates from Brazilian cerrados. An Acad Bras Cienc..

[63] Kuramae EE, Yergeau E, Wong LC, Pijl AS, van Veen JA, Kowalchuk GA (2012). Soil characteristics more strongly influence soil bacterial communities than land-use type. FEMS Microbiol Ecol.

[64] Vierheilig J, Farnleitner AH, Kollanur D, Blöschl G, Reischer GH (2012). High abundance of genetic Bacteroidetes markers for total fecal pollution in pristine alpine soils suggests lack in specificity for feces. J Microbiol Methods.

[65] Lauber CL, Hamady M, Knight R, Fierer N (2009). Pyrosequencing-based assessment of soil pH as a predictor of soil bacterial community structure at the continental scale. Appl Environ Microbiol.

[66] Zhalnina K, Dias R, de Quadros P, Davis-Richardson A, Camargo FO, Clark I, McGrath S, Hirsch P, Triplett E (2015). Soil pH determines microbial diversity and composition in the park grass experiment. Microb Ecol.

[67] Mendes IC, Fernandes MF, Chaer GM, dos Reis Junior FB (2012). Biological functioning of Brazilian Cerrado soils under different vegetation types. Plant Soil.

[68] Miki T, Ushio M, Fukui S, Kondoh M (2010). Functional diversity of microbial decomposers facilitates plant coexistence in a plant–microbe–soil feedback model. Proc Natl Acad Sci USA.

[69] Mitchell RJ, Hester AJ, Campbell CD, Chapman SJ, Cameron CM, Hewison RL, Potts JM (2010). Is vegetation composition or soil chemistry the best predictor of the soil microbial community?. Plant Soil.

[70] Castro AP, Quirino B, Allen H, Williamson L, Handelsman J, Krüger R (2011). Construction and validation of two metagenomic DNA libraries from Cerrado soil with high clay content. Biotechnol Lett.

[71] Fierer N, Leff JW, Adams BJ, Nielsen UN, Bates ST, Lauber CL, Owens S, Gilbert JA, Wall DH, Caporaso JG (2012). Cross-biome metagenomic analyses of soil microbial communities and their functional attributes. Proc Natl Acad Sci USA.

[72] Nicol GW, Leininger S, Schleper C, Prosser JI (2008). The influence of soil pH on the diversity, abundance and transcriptional activity of ammonia oxidizing archaea and bacteria. Environ Microbiol.

[73] Xia W, Zhang C, Zeng X, Feng Y, Weng J, Lin X, Zhu J, Xiong Z, Xu J, Cai Z (2011). Autotrophic growth of nitrifying community in an agricultural soil. ISME J.

[74] Wu Y, Conrad R (2014). Ammonia oxidation-dependent growth of group I.1b *Thaumarchaeota* in acidic red soil microcosms. FEMS Microbiol Ecol.

[75] Castro AP, Quirino B, Pappas G, Kurokawa A, Neto E, Krüger R (2008). Diversity of soil fungal communities of Cerrado and its closely surrounding agriculture fields. Arch Microbiol.

[76] Ma A, Zhuang X, Wu J, Cui M, Lv D, Liu C, Zhuang G (2013). Ascomycota members dominate fungal communities during straw residue decomposition in arable soil. PLoS ONE.

[77] Baldrian P, Kolarik M, Stursova M, Kopecky J, Valaskova V, Vetrovsky T, Zifcakova L, Snajdr J, Ridl J, Vlcek C (2012). Active and total microbial communities in forest soil are largely different and highly stratified during decomposition. ISME J.

[78] Vance E, Brookes P, Jenkinson D (1987). An extraction method for measuring soil microbial biomass C. Soil Biol Biochem.

[79] Williamson KE, Radosevich M, Wommack KE (2005). Abundance and diversity of viruses in six Delaware soils. Appl Environ Microbiol.

[80] Ashelford KE, Day MJ, Fry JC (2003). Elevated abundance of bacteriophage infecting bacteria in soil. Appl Environ Microbiol.

[81] Uroz S, Ioannidis P, Lengelle J, Cébron A, Morin E, Buée M, Martin F (2013). Functional assays and metagenomic analyses reveals differences between the microbial communities inhabiting the soil horizons of a Norway spruce plantation. PLoS ONE.

[82] Yu K, Zhang T (2012). Metagenomic and metatranscriptomic analysis of microbial community structure and gene expression of activated sludge. PLoS ONE.

[83] Delmont TO, Prestat E, Keegan KP, Faubladier M, Robe P, Clark IM, Pelletier E, Hirsch PR, Meyer F, Gilbert JA (2012). Structure, fluctuation and magnitude of a natural grassland soil metagenoma. ISME J.

[84] Lopes AAC, Sousa DMG, Reis Junior FB, Mendes IC (2015). Air-drying and long-term storage effects on β-glucosidase, acid phosphatase and arylsulfatase activities in a tropical savannah oxisol. Appl Soil Ecol.

[85] Embrapa S (1997). Manual de métodos de análise de solo.

[86] Joergensen RG (1996). The fumigation-extraction method to estimate soil microbial biomass: calibration of the *k*_EC_ value. Soil Biol Biochem.

[87] Brookes PC, Landman A, Pruden G, Jenkinson D (1985). Chloroform fumigation and the release of soil nitrogen: a rapid direct extraction method to measure microbial biomass nitrogen in soil. Soil Biol Biochem.

[88] Tabatabai M. Soil enzymes. In: Methods of Soil Analysis: Part 2—Microbiological and Biochemical Properties. Book Series 5. Madison: SSSA; 1994. p. 775-833.

[89] Gomez-Alvarez V, Teal TK, Schmidt TM (2009). Systematic artifacts in metagenomes from complex microbial communities. ISME J.

[90] Overbeek R, Begley T, Butler RM, Choudhuri JV, Chuang H-Y, Cohoon M, de Crécy-Lagard V, Diaz N, Disz T, Edwards R (2005). The subsystems approach to genome annotation and its use in the project to annotate 1000 genomes. Nucleic Acids Res.

[91] Wilke A, Glass EM, Bischof J, Braithwaite D, DSouza M, Gerlach W, Harrison T, Keegan K, Matthews H, Paczian T (2013). MG-RAST Technical Report and Manual for Version 3.3. 6–rev.

[92] Parks DH, Beiko RG (2010). Identifying biologically relevant differences between metagenomic communities. Bioinformatics.

