# Supplementary material for: Shifts in taxonomic and functional microbial diversity with agriculture: How fragile is the Brazilian Cerrado?
Source: BMC Microbiol. 2016 Mar 16;16:42. doi: 10.1186/s12866-016-0657-z (PMC4794851; doi:10.1186/s12866-016-0657-z)
Supplement: Additional file 1: Table S1. — Main features of the compositing metagenomes based on the MG-RAST annotations. Treatments correspond to soils under native vegetation of Cerrado (Native) or cropped with soybean/corn under no-tillage (NT) or conventional tillage (CT) systems. (DOCX 18 kb) [file 12866_2016_657_MOESM1_ESM.docx]

**S1** **Table.** Main features of the compositing metagenomes based on the MG-RAST annotations. Treatments correspond to soils under native vegetation of Cerrado (Native) or cropped with soybean/corn under no-tillage (NT) or conventional tillage (CT) systems

| *Features* | *Metagenome* | | | | | | | | |
| --- | --- | --- | --- | --- | --- | --- | --- | --- | --- |
|  | *NT1* | *NT2* | *NT3* | *CT1* | *CT2* | *CT3* | *Native1* | *Native2* | *Native3* |
| Total number of reads | 5,912,295 | 5,234,816 | 5,708,651 | 5,420,302 | 5,850,205 | 4,898,256 | 5,657,434 | 5,040,252 | 5,460,208 |
| Total number of base pairs | 1,466,364,965 | 1,372,567,727 | 1,507,973,051 | 1,486,805,608 | 1,686,703,041 | 1,303,666,221 | 1,562,052,023 | 1,221,306,956 | 1,470,456,607 |
| Mean sequence length | 248 ± 58 bp | 262 ± 79 bp | 264 ± 68 bp | 274 ± 78 bp | 288 ± 82 bp | 266 ± 92 bp | 276 ± 78 bp | 242 ± 84 bp | 269 ± 80 bp |
| Mean GC contente (%) | 63 ± 7% | 63 ± 8% | 63 ± 8% | 63 ± 8% | 63 ± 8% | 63 ± 8% | 60 ± 6% | 61 ± 6% | 61 ± 7% |
| Ribosomal RNA | 30,702 | 23,298 | 27,374 | 25,119 | 4,441 | 3,710 | 42,110 | 2,524 | 2,850 |
| Annotated proteins | 2,484,162 | 2,221,408 | 2,419,021 | 2,344,760 | 2,515,825 | 1,918,496 | 2,362,338 | 1,888,382 | 2,179,884 |
| *Unknown proteins | 3,213,210 | 2,799,274 | 3,078,065 | 2,866,059 | 3,116,906 | 2,789,980 | 3,052,555 | 2,847,620 | 3,021,744 |
| *Unknown | 184,170 | 190,773 | 184,146 | 184,286 | 0 | 79,806 | 200,417 | 150,537 | 83,107 |
| Failed QC* | 50 | 62 | 44 | 77 | 213,033 | 106,181 | 13 | 151,110 | 172,529 |

* QC= Quality Control (base-call quality filtering, read-length filtering, and de-replication of reads).

* Unknown proteins – predicted proteins but with unknown function

* Unknown – proteins that were confirmed by QC, but have no rRNA genes or do not match predicted proteins.
