# Supplementary material for: Shifts in taxonomic and functional microbial diversity with agriculture: How fragile is the Brazilian Cerrado?
Source: BMC Microbiol. 2016 Mar 16;16:42. doi: 10.1186/s12866-016-0657-z (PMC4794851; doi:10.1186/s12866-016-0657-z)
Supplement: Additional file 2: Figure S1. — Rarefaction curves generated with the MG-RAST software against M5NR database using normalized values between 0 and 1 for no-tillage (NT), conventional tillage (CT) and undisturbed Cerrado (Native) soil metagenomes. Figure S2. Sequence abundance orders of Betaproteobacteria compared to M5NR database using normalized values between 0 and 1 for no-tillage (NT), conventional tillage (CT) and undisturbed Cerrado (Native) soil metagenomes. The order Burkholderiales was the most abundant in the NT system, followed by Nitrosomonadales, both in CT and NT (p < 0.05). Figure S3. Sequence abundance of phyla of Archaea Domain compared to M5NR database, and using normalized values between 0 and 1 for no-tillage (NT), conventional tillage (CT) and undisturbed Cerrado (Native) soil metagenomes. Crenarchaeota was higher in the NT, while Thaumarchaeota and unclassified were higher in the NT and CT treatments (p < 0.05). Figure S4. Sequence abundance of the phyla of Eukaryota Domain compared to M5NR database and using normalized values between 0 and 1 for no-tillage (NT), conventional tillage (CT) and undisturbed Cerrado (Native) soil metagenomes. Figure S5. Sequence abundance in the Viruses domain compared to M5NR database using normalized values between 0 and 1 for no-tillage (NT), conventional tillage (CT) and undisturbed (Native) soil metagenomes. Caudovirales was higher in the NT and CT systems (p < 0.05). (DOCX 423 kb) [file 12866_2016_657_MOESM2_ESM.docx]

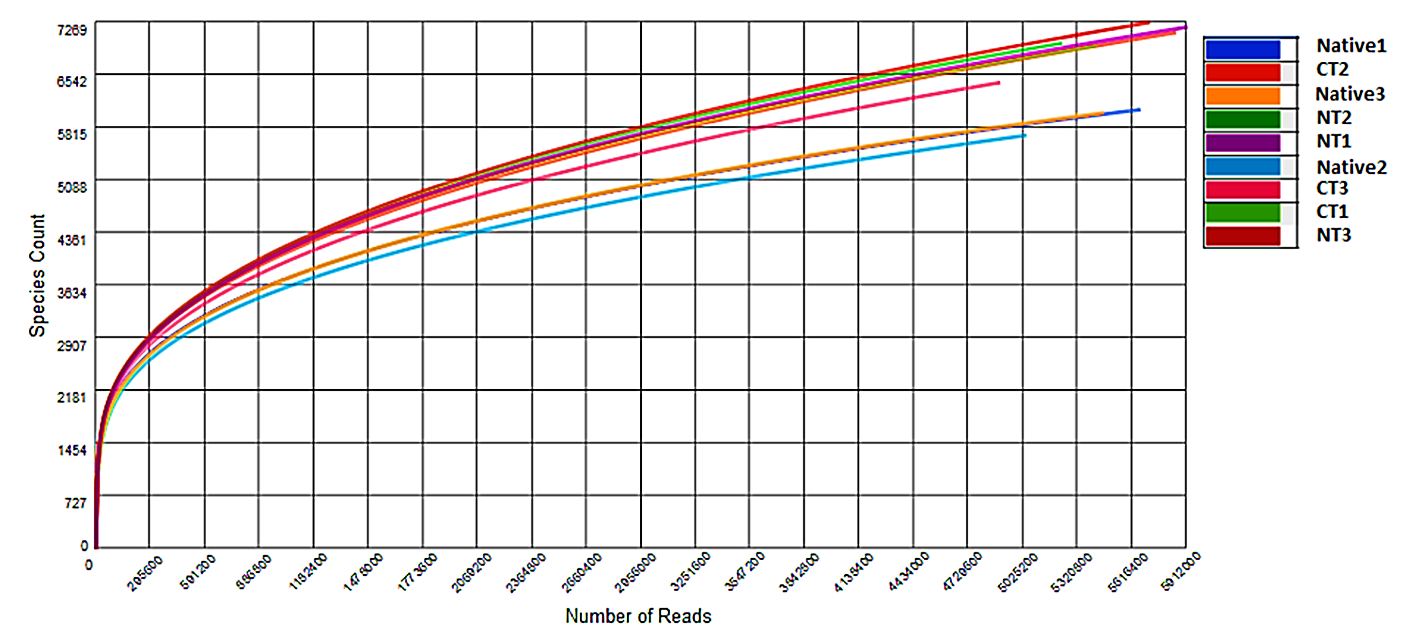


**S1** **Fig.** Rarefaction curves generated with the MG-RAST software against M5NR database using normalized values between 0 and 1 for no-tillage (NT), conventional tillage (CT) and undisturbed Cerrado (Native) soil metagenomes.

**S2 Fig.** Sequence abundance orders of Betaproteobacteria compared to M5NR database using normalized values between 0 and 1 for no-tillage (NT), conventional tillage (CT) and undisturbed Cerrado (Native) soil metagenomes.

**S3** **Fig.** Sequence abundance of phyla of Archaea Domain compared to M5NR database, and using normalized values between 0 and 1 for no-tillage (NT), conventional tillage (CT) and undisturbed Cerrado (Native) soil metagenomes.

**S4** **Fig.** Sequence abundance of the phyla of Eukaryota Domain compared to M5NR database and using normalized values between 0 and 1 for no-tillage (NT), conventional tillage (CT) and undisturbed Cerrado (Native) soil metagenomes.

**S5 Fig.** Sequence abundance in the Viruses domain compared to M5NR database using normalized values between 0 and 1 for no-tillage (NT), conventional tillage (CT) and undisturbed (Native) soil metagenomes.
